# Primary anastomosis versus stoma following urgent sigmoidectomy for sigmoid volvulus: 58-year experience in a tertiary referral center

**DOI:** 10.12669/pjms.40.11.10543

**Published:** 2024-12

**Authors:** Enes Agirman, Esra Disci, Rifat Peksoz, Sabri Selcuk Atamanalp

**Affiliations:** 1 Enes Agirman, MD. Assistant Professor, Department of General Surgery, Erzurum City Hospital, Erzurum, Turkiye; 2 Esra Disc, MD. Associate Professor, Faculty of Medicine, Department of General Surgery, Ataturk University, Erzurum, Turkiye; 3 Rifat Peksoz, MD. Associate Professor, Faculty of Medicine, Department of General Surgery, Ataturk University, Erzurum, Turkiye; 4 Sabri Selcuk Atamanalp, MD Professor, Faculty of Medicine, Department of General Surgery, Ataturk University, Erzurum, Turkiye

**Keywords:** Sigmoid volvulus, Sigmoidectomy, Intestinal continuity, Primary anastomosis, Stoma

## Abstract

**Objectives:**

Primary anastomosis and stoma are the main options in the restoration of intestinal continuity following urgent sigmoidectomy in sigmoid volvulus (SV). Our purpose was to evaluate the outcomes of both techniques in a 1,083-patient SV series.

**Methods:**

Total 1,083 cases with SV treated in Ataturk University Research Hospital in 58-year period between June 1966 and July 2024 were included in this study. We reviewed the records of 612 patients (56.5%) retrospectively, while the remaining 471 cases (43.5%) were evaluated prospectively. We investigated some preoperative, operative, and postoperative characteristics in non-matched analyses.

**Results:**

Among total 379 patients treated with urgent colectomy, primary anastomosis was used in 173 cases (45.6%), while stoma was required in 206 patients (54.4%). The mean age was significantly lower in primary anastomosis group (P<0.005), while male/female ratios were statistically similar (P>0.05). Mean ASA score (P<0.001) and rates of shock (P<0.001), bowel gangrene (P<0.001), bowel perforation (P<0.01), and risky bowel (P<0.005) were also significantly lower in the primary anastomosis group. When stoma closure was considered, operation time was significantly shorter (P<0.001), additionally, morbidity and mortality rates were significantly lower in the primary anastomosis group (P<0.001, in each). The distributions of reoperation rates were statistically similar in both groups (P>0.05). Conversely, hospitalization time was significantly shorter and cost was significantly lower in the primary anastomosis group (P<0.001, in each).

**Conclusion:**

Primary anastomosis has some advantages in comparison to stoma in the restoration of intestinal continuity following urgent sigmoidectomy in SV. However, stoma is generally preferred in patients with bad health status, old age, and risky bowel. New prospective randomized clinical studies or matched analyses may help to clarify the optimal choice.

## INTRODUCTION

In sigmoid volvulus (SV), endoscopic detorsion is the first-line treatment in uncomplicated patients, while urgent surgery is an inevitable ending in cases with peritonitis, bowel gangrene or perforation, and unsuccessful endoscopy.^1^,^2^ In urgent surgery, although detorsion alone or a recurrence-reducing procedure such as sigmoidopexy, mesopexy, mesoplasty, or even sigmoidectomy are the main alternatives in patients with viable sigmoid colon, sigmoidectomy is necessary in cases with gangrenous bowel.^3^,^4^ Following sigmoidectomy, nonelderly patients with good general status are the main candidates for primary anastomosis in the restoration of intestinal continuity.^5^

Conversely, bad general status and old age in addition to local risk factors including bowel perforation, peritonitis, borderline ischemia, edema, or different diameter in bowel ends force the practitioners to perform a stoma as a traditional approach.^6^ However, there is limited data on the comparison of primary anastomosis and stoma in decision-making process.^7^ In this article, to evaluate the roles of primary anastomosis and stoma in the restoration of intestinal continuity following sigmoidectomy, we utilized the surgical outcomes of 444 patients among 1,083 SV cases treated over 58 years (from June 1966 to July 2024), which data constitutes the largest single-center SV series over the world.^8^

## METHODS

One thousand eighty three cases with SV treated in Ataturk University Research Hospital in 58-year period between June 1966 and July 2024 were included in this study. In 612 (56.5%) patients, who were treated between June 1966 and June 1986, the hospital records were reviewed retrospectively, while 471 cases (43.5%) treated between June 1986 to July 2024, were evaluated prospectively. For each patient, preoperative (age, gender, American Society of Anesthesiologists-ASA score, and shock), operative (bowel gangrene, perforation, local risk factors such as borderline ischemia, edema, or different diameter in bowel ends, and operation time), and postoperative data (mortality, morbidity, reoperation, hospitalization time, and cost) were recorded.

In the treatment of SV, nonsurgical detorsion (principally endoscopy) was tried in uncomplicated patients (without peritonitis and bowel gangrene or perforation), additionally, elective sigmoidectomy was performed in selected cases (nonelderly individuals with good general status) with successful nonsurgical detorsion. Patients with above-mentioned poor conditions or unsuccessful nonsurgical detorsion were treated with urgent surgery. In surgery, sigmoidectomy was performed in all cases with bowel gangrene and in some selected patients with viable bowel. To restore the intestinal continuity, primary anastomosis (with or without bowel cleansing) was generally performed in nonelderly patients with good general status, while stoma (Hartmann’s procedure or Mikulicz procedure) was applied in most elderly cases with poor general status. Additionally, patients with bowel perforation, peritonitis, borderline ischemia, edema, or different diameter in bowel ends were generally treated with stoma. In operative colonic cleansing, following the distal resection of the sigmoid colon, we applied 3,000 cc. isotonic sodium chloride solution via tube cecostomy from the base of the appendix. In the final evaluation, we compared the results of primary anastomosis group with that of stoma group.

### Statistical Analysis:

SPSS software (Version 22, SPSS Inc, Chicago, IL, USA) was used in statistical analysis of the data. Descriptive results for categorical variables were reported as numbers and percentages. We used Chi-square test in the comparison of proportions between categorical variables. Descriptive statistics for numerical data were reported as mean ± standard deviation. In the assessment of the normality of the distribution of numerical data, we used Kolmogorov-Smirnov test and various graphical approaches (histograms and Q-Q plots). To evaluate the homogeneity of variances between groups, we preferred Levene’s test. Student’s t-test was employed to compare numerical data between groups, based on the assumptions of parametric tests. A significance level of P<0.05 was set for statistical significance.

### Ethical Approval:

For this study, ethical approval was obtained from institutional review board (Ataturk University Faculty of Medicine, Ethical Committee, 21.02.2024/68). Informed consents were obtained from the patients or their caregiver family members.

## RESULTS

We applied urgent surgery in 488 (45.1) of total 1,083 patients with SV, 379 of which (77.7%) treated with sigmoidectomy. In the restoration of intestinal continuity, we used primary anastomosis in 173 patients (45.6% of total, with operative colonic cleansing in 76, without this procedure in 97), while stoma was required in 206 cases (54.4% of total, Hartmann’s procedure in 192, Mikulicz procedure in 14). The preoperative, operative, and postoperative characteristics of 379 patients are presented in Table-I.

**Table-I:** Preoperative, operative, and postoperative characteristics of patients with sigmoid volvulus.

Parameter	Primary anastomosis	Stoma	Statistical analysis
Total	173	206	-
Age	56.8	61.2	t=2.87, P=0.004^d^
Gender (male/female) % ratio	141/32	169/37	X=0.018, P=0.893^e^
	81.5/18.5	82.0/17.0	
	4.4	4.6	
ASA score	3.0	3.4	t=5.419, P<0.001^d^
Shock %	10	84	X=61.748, P<0.001^e^
	5.8	40.8	
Bowel gangrene %	114	171	X=14.766, P<0.001^e^
	65.9	83.0	
Bowel perforation %	2	13	X=6.573, P=0.01^e^
	1.2	6.3	
Risky bowel^a^%	8	28	X=8.797, P=0.003^e^
	4.6	13.6	
Operation time^b^ (minute)	189.5	161.6	t=4.491, P<0.001^d^
Operation time^c^ (minute)	189.5	222.7	t=4.68, P<0.001^d^
Mortality^b^%	22	50	X=8.159, P=0.004^b^
	12.7	24.3	
Mortality^c^%	22	56	X=12.042, P<0.001^e^
	12.7	27.2	
Morbidity^b^%	58	83	X=1.842, P=0.175^e^
	33.5	40.3	
Morbidity^c^%	58	113	X=17.275, P<0.001^e^
	33.5	54.9	
Reoperation^b^%	13	14	X=0.073, P=0.787^e^
	7.5	6.8	
Hospitalization time^b^ (day)	11.8	15.6	t=4.703, P<0.001^d^
Hospitalization time^c^ (day)	11.8	25.2	t=12.727, P<0.001^d^
Cost^b^ (USD)	3,058.5	3,669.6	t=5.586, P<0.001^d^
Cost^c^ (USD)	3,058.5	4,856.4	t=15.761, P<0.001^d^

ASA: American Society of Anesthesiologists,

a : borderline ischemia, edema, or different diameter in bowel ends;

b : excluding stoma closure,

c : including stoma closure,

d Student’s t-test,

e Chi-square test.

Preoperative data of our series presented that, the mean age of the primary anastomosis group was significantly lower than that of the stoma group (56.8 years vs. 61.2 years, P<0.005), while male/female ratios were statistically similar (4.4 vs. 4.6, P>0.05). However, mean ASA scores (3.0 vs. 3.4, P<0.001) and rate of shock (5.8% vs. 40.8%, P<0.001) were significantly lower in the primary anastomosis group compared to the stoma group.

Operative data of the present series showed that, rates of bowel gangrene (65.9% vs. 83.0%, P<0.001), bowel perforation (1.2% vs. 6.3%, P<0.01), and risky bowel (4.6% vs. 13.6%, P<0.005) were significantly lower in the primary anastomosis group compared to the stoma group. When excluding the stoma closures, operation time was significantly longer in the primary anastomosis group compared to the stoma group (189.5 minutes vs. 161.6 minutes, P<0.001), while it was significantly shorter in the primary anastomosis when including the stoma closures (189.5 minutes vs. 222.7 minutes, P<0.001).

Postoperative evaluation of our series presented that, mortality rates were significantly lower in the primary anastomosis group compared to the stoma group in both excluding (12.7% vs. 24.3%, P<0.005) and including (12.7% vs. 27.2%, P<0.001) the stoma closures. Morbidity rates were not significantly different when excluding the stoma closures (33.5% vs. 40.3%, P>0.05), while it was significantly lower in the primary anastomosis group when including the stoma closures (33.5% vs. 54.9%, P<0.001). The main complications were wound infection (12.6% vs. 31.2%), pulmonary atelectasis or embolism (5.8% vs. 12.1%), myocardial infarction (2.4% vs. 4.6%), leakage from anastomosis or stoma (4.9% vs. 1.7%), paralytic ileus (3.9% vs. 2.3%), evisceration (1.5% vs. 4.0%), renal insufficiency (1.0% vs. 0.6%), internal herniation (0.0% vs. 0.6%), and disseminated intravascular coagulation (0.0% vs. 0.6%) in the primary anastomosis and stoma groups, respectively. The distributions of reoperation rates between the groups were statistically similar (7.5% vs. 6.8%, P>0.05). Conversely, hospitalization times were significantly shorter in the primary anastomosis group when compared with that of the stoma group in both excluding (11.8 days vs. 15.6 days, P<0.001) and including (11.8 days vs. 25.2 days, P<0.001) the stoma closures. Similarly, primary anastomosis operations were significantly less costly in comparison to stoma operations in both excluding (3,058.5 USD vs. 3,669.6 USD, P<0.001) and including (3,058.5 USD vs. 4,856.4 USD, P<0.001) the stoma closures.

## DISCUSSION

In SV, sigmoidectomy is the sole treatment option in cases with bowel gangrene. Additionally, it is a good solution in most non-gangrenous cases.^9^ SV recurs in 0%-85% (mean: 25%) of all cases. Among several factors, age (elderliness), onset (early onset), gender (maleness), diet habits (high-fiber diet), living conditions (living in high altitude), and defecation habits (chronic constipation) are the main agents affecting SV recurrence.^10^,^11^ Sigmoidectomy extinguishes dolichosigmoid, the main anatomical predisposition of both primary and recurrent SV.^12^,^13^ Following sigmoidectomy, in order to achieve the restoration of intestinal continuity, there are principally two ways including primary anastomosis and stoma. However, there is limited data to guide decision-making process.^14^

Despite some worse preoperative (older mean age, higher mean ASA scores, and higher rate of shock) and operative data (higher rates of bowel gangrene, perforation, and risky bowel) in stoma group, our results demonstrated shorter mean operation time, lower rates of mortality and morbidity, shorter mean hospitalization time, and less costly in primary anastomosis group.

Among a number of retrospective reports comparing primary anastomosis and stoma following sigmoidectomy in SV, Awedew et al.^15^ reported non-significant lower mortality (15% vs. 19%) and higher morbidity rates in primary anastomosis group in a 724-case study. Likewise, Mulugeta and Awlachew^16^ presented similar mortality and morbidity rates (6.4% vs. 5.6% and 43.8% vs. 33.3%, respectively) in primary anastomosis and stoma groups, in their 96-patient urgent surgery series. Similarly, comparable mortality rates (33.3% vs. 30.0%) were reported by Rajsiddharth et al.^17^ in a 16-case study. In furtherance of these findings, Kuzu et al.^18^ demonstrated a leakage rate of 7% with a mortality rate of 6.6% across both groups in a study of 106 SV cases.

However, Pattanaik^19^ demonstrated higher mortality rate (19.2% vs. 7.5%) in stoma group in 362 surgically treated patients. Similarly, a relatively higher mortality rate (26.8% vs. 18.2%) was reported by Maddah et al.^20^ in stoma group in a 16-case study. Correlatively, higher infectious morbidity rate (21.5% vs. 6.6%) was found in a 62-patient report by Traore et al.^21^ In most of the above-mentioned studies similar to our results, the main complications were wound infection, pulmonary, cardiac, and renal problems, ileus, and leakage from anastomosis or stoma. In our series, both mortality and morbidity rates were statistically higher in stoma group, similar to some of above-mentioned reports. Additionally, mean operation time and hospitalization time were longer and cost was higher in patients treated with stoma.

However, as a traditional clinical approach, most surgeons avoid medico-legal responsibilities and use primary anastomosis in well-conditioned and nonelderly patients, while they prefer stoma in bad-conditioned and elderly cases and those with risky bowel.^6^,^22^ Our non-matched analyses on preoperative data support this practice. For this reason, matched statistical evaluation becomes crucial at this point.

Among limited data comparing primary anastomosis and stoma following sigmoidectomy by using matched statistical evaluation, Yang et al.^7^ presented similar mortality (12.5% vs. 10.5%), morbidity (41.7% vs. 38.5%), and readmission rates (12.4% vs. 13.3%) in primary anastomosis and stoma groups, respectively, in a multicenter 895-case retrospective study. Additionally, they found similar length of hospital stay (11.3 days vs. 11.8 days) in both groups. However, they did not consider the total hospitalization time including stoma closure operation, economic burden arising from second operation and stoma care materials, and quality of life, which were the main discussion points of their study.

In another multicenter matched evaluation, Dolejs et al.^23^ reported similar mortality (10.8% vs. 12.1%), morbidity (53.0% vs. 53.9%), and readmission rates (16.0% vs. 11.6%) in primary anastomosis and stoma groups, respectively, in 464-patient matched evaluation. However, length of hospital was significantly longer in stoma group (7 days vs. 9 days) in this study. As is well known, stoma requires a second procedure to restore the intestinal continuity, which has additional mortality and morbidity in itself, and causes prolonged operation time and cost in addition to impaired quality of life.^22^,^24^ However, it should not be forgotten that, stoma may be indispensable and life-saving in selected cases.^4^,^6^,^25^

As a crucial point, our experiments demonstrated that, primary anastomosis appears as a favorable option in the restoration of intestinal continuity following urgent sigmoidectomy in selected patients with SV. However, stoma may save life in some others. New prospective randomized clinical studies or matched analyses may add new information about the treatment algorithm.

### Limitations:

Nonrandomized grouping, partial retrospective evaluation of the data, and non-matched statistical analysis are the main limitations of this study. However, large-series prospective studies require tens of patients and decades. Instead, matched analyses of retrospective data may give additional information.

## CONCLUSIONS

Similar to our results, some non-matched retrospective studies also demonstrate better postoperative outcomes by using primary anastomosis instead of stoma in the restoration of intestinal continuity following urgent sigmoidectomy in SV. However, some other matched reports do not favour this idea. As such new prospective randomized clinical studies or matched analyses may clarify the current algorithm.

### Authors’ Contribution:

**EA:** Data collection, manuscript writing.

**ED and RP:** Data collection, revision of the final draft.

**SSA:** Data collection, manuscript writing.

All authors have read the final manuscript and are responsible and accountable for the accuracy or integrity of the work.
